# Supplementary material for: Are carbon emissions trading and green financial instruments synergistic? -Comprehensive quantitative research based on content analysis
Source: PLoS One. 2024 Mar 7;19(3):e0298601. doi: 10.1371/journal.pone.0298601 (PMC10919601; doi:10.1371/journal.pone.0298601)
Supplement: S1 Data — (ZIP) [file pone.0298601.s001.zip › Data/地级市Z_F工作报告.zip!/╔╜╬≈/╠1⁄2╘¡╩╨/└┤╫╘╣1⁄2╓┌║┼í╛┬φ┐╦╩2╛▌═°í┐_2003.docx]

各位代表：

现在，我代表市政府向大会作报告，请予审议，并请各位政协委员和其他列席人员提出意见。

一、2002年工作回顾

2002年是我市率先发展的起步之年。一年来，全市人民高举邓小平理论伟大旗帜，忠诚实践“三个代表”重要思想，在省委、省政府和市委的正确领导下，开拓创新，团结奋进，较好地完成了市十一届人大一次会议确定的各项目标任务。全市经济快速健康发展，主要指标实现了两位数增长，创“九五”以来最好水平。全年完成国内生产总值433.6亿元，增长13.5%；财政总收入46.7亿元，增长21.6%（包含两个省直税务分局，财政总收入为72.5亿元，增长12.9%）；城镇居民人均可支配收入7376元，增长13.5%；农民人均纯收入3077元，增长12.4%。实现了率先发展的良好开局。

结构调整初见成效。去年扶持的41个市属潜力产品项目，90%建成投产、70%达产见效；列入省“1311”规划的71个潜力产品项目，71%投产。潜力产品企业实现工业产值204亿元，拉动全市工业增长11.7个百分点。产品、产业和所有制结构发生趋势性变化。传统产业改造提升，产业链延伸，附加值增加，效益逐步显现；生物制药、电子信息、新材料等新兴产业初具雏形，发展势头强劲。非公有制经济活力日益增强，占GDP的比重提高到35%，对财政的贡献率达到20%。结构调整三年初见成效的目标基本实现。

工业经济稳步增长。全市规模以上工业总产值完成380.4亿元，增长16.3%；工业增加值完成115亿元，增长15%；实现利税31.9亿元，增长18.2%。工业经济效益综合指数82.2，比上年提高6.3。国有企业兼并、破产、重组和投资主体多元化改革逐步加快。高新区、经济区、民营区发展健康，正在成为全市新的经济增长极。高新区国内生产总值、财政总收入分别比上年增长51.9%和63.8%。经济区完成国内生产总值和税收分别增长120%和31%。民营区工业总产值增长1.3倍。

城市建设力度加大。完成固定资产投资147.6亿元，增长20.3%。黄河水源城市供水、二电集中供热、胜利桥加宽、和平北路、南内环桥西引道、柴西路、迎春街、城坊东街、滨河东路向北延伸南段、旧晋祠路南段拓宽改造等一批重点工程建成投运，新增集中供热能力600万平方米，新增煤气供气能力1.1亿立方米。各类房产竣工263万平方米，房产交易额39亿元。经营城市取得新突破，国土纯收益5.6亿元，广告经营权拍卖成交额1.3亿元。公路网建设步伐加快，增加水泥、沥青路面250公里。

城市环境进一步改善。大力开展城市环境综合整治，拆除违建临建310万平方米，取缔马路市场185个，新建规范蔬菜市场90个，铺装便道115万平方米，整饰建筑立面671万平方米，新增绿地317万平方米，对32条主干道、出口路进行了整治和美化亮化，改造了20个交通路口。迎泽公园免费开放，新建了一批贴近市民的广场和游园，绿化覆盖率达到33.7%，绿地率26.8%，人均绿地7平方米。汾河景区荣获“迪拜国际改善人居环境最佳范例奖”。市区空气质量持续改善，二级以上天数达到153天，主要污染物指标平均下降15%以上。生态建设迈出新步伐，环城林带完成7.8万亩。城市规划进一步加强，编制了文物古迹保护规划等25个专业性规划和6个分区规划。

农村经济健康发展。农林牧渔业总产值完成26.9亿元，增长14.2%。农业结构日趋优化，粮经比由上年的7：3调整为6.4：3.6，畜牧业产值占农业总产值的比重上升到37.4%。农业产业化龙头企业发展到110个，完成农产品加工产值20亿元。退耕还林44.7万亩。乡镇企业完成增加值56.6亿元，增长30.6%。坚持开发式扶贫方针，解决了10万人的饮水困难，2.9万人脱贫。

商贸、旅游业持续增长。社会消费品零售总额完成181亿元，增长12.3%。做好“假日经济”文章，“五一”、“十一”期间，商品销售额比平日增长16%。大力发展新型业态，新增超市、连锁便利店134个。加快旅游业发展，对晋祠景区、神堂沟温泉旅游度假区进行了综合整治，全年接待海内外游客1043万人次，旅游总收入35.7亿元，增长16.1%。

对外开放态势良好。地区进出口总额完成16.4亿美元，增长24.2%。又有30户企业获得对外贸易经营权。出口商品结构发生积极变化，机电产品出口额增长21.1%。成功举办了太原国际经济合作论坛暨投资项目洽谈会和投资项目香港推介活动，签约项目179个，协议外资19亿美元。新批外商投资企业32户，投资总额2亿美元，增长1.4倍；合同利用外资1.2亿美元，增长2.6倍。外事工作向多领域发展，对外经济文化交流不断扩大。

财税运行质量提高。完善“金税工程”，加强税源监控，各项税收实现了均衡入库、稳定增长。优化支出结构，确保了法定支出、重点支出的需要。部门预算、国库集中支付、政府采购等改革不断深化。调整了市对县区的财政管理体制。全市金融健康运行，对经济建设的支持力度进一步加大。

社会事业改革推进。“普九”成果巩固提高，小学生流失率、初中生辍学率控制在0.5%和1.5%以内。理顺义务教育管理体制，完成了14所初中下放城区和8所市属企业学校的剥离。城区基本普及了高中阶段教育，高考升学率全省领先。发挥科技在调整结构中的先导作用，我市被确定为国家和省制造业信息化工程重点城市。健全四级农业技术推广服务体系，对12万人次农民进行了科技培训。深化文化事业改革，组建了广播电视总台、电影、演艺集团。成功举办了首届晋阳文化艺术节。整合卫生资源，组建了中心医院、妇幼保健、中医药等3个医疗集团。积极推进农村卫生工作，不断满足农民的基本卫生服务需求。扩大社区卫生服务覆盖面，受益人口150余万。开展全民健身运动，承办了第11届省运会，夺得金牌总数和总分第一名。人事制度改革不断深化，公开招考录用公务员工作迈出新步伐。文物保护工作不断加强，王家峰北齐墓发掘工作全面完成，晋阳古城遗址规划保护被联合国开发署确定为城市规划援助项目。社区建设水平进一步提高，社区干部结构得到优化。首批129个“星光计划”项目顺利建成，社区功能不断增强，荣获“全国社区建设示范城市”称号。

社会保障体系日益完善。安置下岗职工1.85万人，再就业率42%。养老保险、失业保险覆盖面达到95%，社会化发放率保持100%。为2.7万名国有企业下岗职工按时足额发放基本生活费、代缴社会保险费1.2亿元。为20.5万名企业离退休人员发放基本养老金13.8亿元。为3.1万名退休人员补发了历年拖欠的养老金。为1.9万名失业人员发放失业保险金4726万元。城镇职工基本医疗保险参保50万人。城市居民最低生活保障8.5万人，发放低保金5730万元，做到了应保尽保，并初步实现了社会化发放。完善自然灾害救助体系，救济受灾群众5万余人，发放救济金748万元，发放救灾粮261万公斤。残疾人工作领域拓宽，就业保障金突破1000万元。

发展环境逐步优化。积极转变政府职能，深化审批制度改革，累计减少审批、收费项目488项，精简幅度为57%；规范审批行为，审批环节、审批时间均减少65%。政务大厅建成投运，对32个部门、179个事项进行集中审批和办理。推行“阳光政务”，政府各部门向社会公开了职能职责和办事程序，开通了政府公众信息网站，扩大了政务公开范围和渠道。建立重大决策项目公示制度和公众参与制度，召开了煤气价格和征收城市生活垃圾处理费听证会。积极推进事权下放，47个市直部门向县区下放事权228项。完善集中收费制度，取消了机关、事业单位的预算外账户，实行“收支两条线”管理。强化行政效能监督，设立了效能监督投诉中心和监督网站，受理投诉902件，有841件得到及时处理。加强廉政建设，加大了源头治理力度，行风“双评”工作不断引深。强化审计监督，对26个项目、523个单位进行了审计和审计调查，对186名干部进行了经济责任审计。落实执法过错和错案责任追究制度，行政执法水平有了新提高。

精神文明和民主法制建设成效显著。深入开展群众性精神文明创建活动，涌现出一批文明社区、文明村镇、文明单位，荣获“全国创建文明城市活动先进城市”称号。自觉接受人大和政协的监督，人大代表、政协委员的议案、提案和建议办复率保持100%。企务、村务公开不断深化，基层民主进一步加强。认真接待群众来信来访，及时化解和处理矛盾。国防后备力量建设力度加大，双拥工作扎实推进，军政军民关系更加巩固。“四五”普法教育深入开展，全民法制观念逐步增强。加强社会治安综合治理，以“打黑除恶”为龙头，严厉打击黑恶势力犯罪团伙和“法轮功”等邪教组织。深入开展“扫黄打非”，扫除社会丑恶现象。开展了“人民调解年活动”，减少了由民间纠纷引发的刑事、治安案件。严格落实安全工作责任制，大力开展专项整治，安全生产呈现平稳态势。加强计划生育服务与管理，人口自然增长率控制在5.6‰。改革统计管理制度，统计工作的监督、预测、服务功能进一步发挥。妇女、儿童和老龄事业健康发展。民族宗教工作不断强化。新闻出版、档案、气象、地震、侨务、对台、人防、地方志等项工作也取得了新成绩。

回顾一年来的工作，我们深深体会到，只要我们坚持以“三个代表”重要思想为指导，解放思想，与时俱进，改革创新，就一定能够开创现代化建设的新局面；只要我们坚持把发展作为执政兴市的第一要务，以率先发展的目标凝聚人心，鼓舞干劲，各项工作就一定能够取得突破性进展；只要我们坚持以民为本，把提高人民生活水平作为政府工作的根本目的，执政为公，勤政为民，就一定能够调动人民群众的积极性和创造性，获得大步前进的不竭动力；只要我们坚持从严治政，转变职能，改进作风，提高效率，改善服务，就一定能够创造良好的环境，激发经济社会发展的旺盛活力。

各位代表，过去的一年，是我市经济持续快速增长，率先发展开局良好的一年；是改革开放全面推进，两个文明建设取得丰硕成果的一年；是城市环境变化较大，人民群众得到较多实惠的一年。这是省委、省政府和市委正确领导的结果，是市人大、市政协监督、支持的结果，是全市人民奋力拼搏的结果。在此，我代表市政府，向全市工人、农民、知识分子和各级干部，向人大代表、政协委员、各民主党派、工商联、人民团体、无党派爱国人士，向驻并部队、武警官兵，向所有关心、支持太原发展的海内外朋友们，致以崇高的敬意和衷心的感谢！

我市经济和社会发展形势总体是好的，正在步入一个新的快速发展期。但是，我们也清醒地看到，在加快发展的进程中，还存在一些不容忽视的困难和问题。主要是：经济增长的内在动力仍显不足，高新技术产业和新型产业还没有成为全市经济的骨干力量，传统产业改造提升的任务还很重；国有企业产权制度改革进展不快，尤其是市属企业规模不大、结构不合理、活力不强的问题没有真正解决；有效需求不足，价格指数持续走低，制约经济健康发展；就业压力较大，扩大就业渠道、保障困难群体生活的任务非常艰巨，有些群众的生活还很困难；群众和投资者对发展环境还有不少意见，市场秩序不够规范，社会诚信度不高，政府职能转变还不到位，工作效率有待继续提高；生态建设、环境保护工作任务非常艰巨，还需要进一步加强。这些问题，我们将高度重视，采取有力措施，认真加以解决。

二、2003年政府工作总体要求和奋斗目标

2003年是深入贯彻落实十六大精神的第一年，也是我市实现率先发展的关键之年，又逢太原建城2500年。我们面临重要的机遇，也面临严峻的挑战。从全国、全省看，地区间综合实力竞争日趋激烈，呈现出百舸争流、千帆竞发的态势。市委审时度势，提出了“一年一个样，三年大变样”，主要经济指标在全省争先的新要求。这是发挥省会城市作用、建设中西部经济强市的必然选择。我们要抢抓机遇，狠抓落实，乘势而上，再攀高峰。

党的十六大描绘了全面建设小康社会的宏伟蓝图。省十届人大一次会议提出全省提前3年实现国内生产总值比2000年翻一番。市八次党代会提出，到2005年国内生产总值达到600亿元，争取提前4年实现国内生产总值比2000年翻一番。从“十五”头两年经济运行情况和发展预测分析，到2006年全市国内生产总值将突破700亿元，提前4年翻番的目标完全可以实现。

根据新的形势和任务，今年政府工作的总体要求是：以邓小平理论和“三个代表”重要思想为指导，认真贯彻落实党的十六大精神，按照省委、省政府和市委的决策部署，把发展作为第一要务，以结构调整为主线，走新型工业化道路；以延伸城市环境整治为龙头，提升城市化水平；以“招商旅游年”和纪念建城2500年为契机，加快对外开放步伐；以增加城乡居民收入为重点，着力改善人民群众的生活质量，力争各项经济指标达到全省领先水平，为建成中西部经济强市、建设生态园林城市、全面建设小康社会奠定坚实基础。

主要预期目标是：国内生产总值达到500亿元，增长15%；财政总收入84亿元，增长16%（不含省直分局收入55.1亿元，增长18%）；一般预算收入29.8亿元，增长15%；固定资产投资规模180亿元，增长22%；规模以上工业增加值134亿元，增长16%；社会消费品零售总额205亿元，增长13%；地区外贸出口总额13亿美元，增长13%；城镇居民人均可支配收入达到8260元，增长12%；农民人均纯收入达到3446元，增长12%；人口自然增长率控制在6‰以内；城镇登记失业率控制在4.5%以内。

上述目标是预测性的，是综合分析今年发展的需要和可能，按照既积极奋进、又切实可行的原则提出来的。只要我们准确把握一切有利条件，聚精会神搞建设，一心一意谋发展，万众一心，团结奋斗，我们的目标就一定能够实现，全市的综合实力和人民群众的生活水平就一定能够迈上一个新台阶。

三、2003年政府工作的主要任务

新的一年，要在统筹兼顾、全面推进的基础上，重点抓好以下几方面工作。

（一）推进产业结构调整，走新型工业化道路

走新型工业化道路是在新的历史条件下，加快工业化和现代化进程的必然选择。我们要牢固树立“工业强市”的思想，把工业放在突出位置来抓，坚持以信息化带动工业化，积极探索具有太原特色的新型工业化路子，重振老工业城市的雄风。

加快产业、产品结构调整步伐。以信息化、集约化、规模化为方向，加快发展生物制药、电子信息、新型材料等高新技术产业，建立健全风险担保机制，促进高新技术成果转化，把高新技术产业早日培育成我市的支柱产业。积极用高新技术改造提升传统产业，实施50项技改项目，申报一批国家“双高一优”项目。抓好信息化关键技术攻关及应用工程，发展一批具有太原特色的信息化关键技术。发挥比较优势，大力培育和发展重矿机械、精密机械、铁路运输机械和机电一体化等制造业，努力打响“太原制造”品牌。改革对潜力产品的扶持方式，选择技术先进、成长性好、市场空间大、竞争力强的大项目，集中财力重点扶持，抓好已投产潜力产品的达产达效，着力培育一批优势产品和优势企业，使企业规模、产品档次、技术水平上一个新台阶。

深化国有企业产权制度改革。通过改制、改组、兼并、破产、拍卖、租赁、转让、下放等方式，加快国有企业改革重组步伐，鼓励国有企业用优良资产吸引、嫁接、置换外资和民间资本，加速推进产权多元化。县区今年力争完成所属企业的改制任务。市属企业改革要取得突破性进展。要充分利用国家主辅分离的优惠政策，推进国有企业主业精干化、辅业专业化和后勤服务社会化。加快建立企业退出机制，抓紧做好已实施破产企业的善后工作。进一步完善国有资产管理委员会、国有资产运营机构、企业三个层次的国有资产管理体制。加强国有、集体企业产权交易管理，防止国有资产流失。支持有条件的企业上市融资。

充分发挥“两区”龙头带动作用。促进工业向高新区和经济区转移集聚，尽快形成产业规模优势。加快“两区”体制、机制、管理、环境创新，进一步明确发展定位，突出各自特色，良性竞争，互动发展。高新区要以发展高新技术产业为重点，积极推进服务环境国际化、招商引资规模化、高新技术产业化、资源利用集约化，主要经济指标力争增长50%。经济区要坚持以工业项目为主、吸收外资为主、出口为主和致力于发展高新技术产业的方针，积极引进大企业、大项目，主要经济指标力争翻一番。民营区也要加快发展，在完善基础设施、优化投资环境、创新运行机制上下功夫，主要经济指标力争增长60%。

大力发展非公有制经济。坚持放心、放胆、放宽、放活、放开的原则，在政治上给鼓励、政策上给优惠、服务上给方便、发展上给支持、法律上给保护，大力推进民营经济健康发展；凡国有、集体资本退出的领域，都支持和鼓励民营经济大胆进入。重点扶持纳税百万元以上、特别是纳税500万元以上非公有制企业的发展，努力培育销售5亿元以上、利税亿元以上的大型民营企业。鼓励、引导民营企业发展高新技术和先进适用技术，推进产权制度改革和公司制改造，加速向科技型、外向型、规模型转变，掀起民营经济发展的新高潮，对全市经济的贡献要有新突破。

（二）实施扩容提质战略，增强城市综合服务功能

坚持高起点规划、高标准建设和高效能管理，进一步拓展发展空间，改善人居环境，提升城市的品位。

强化规划的龙头作用。科学确定城市定位，调整优化城市布局，突出太原古城特色，搞好城市形象设计，完善城市规划体系。论证通过教育、旅游等专业规划，力争编制完成覆盖全市的控制性详细规划、城市发展战略规划、历史文化名城保护规划、近期建设规划和汾河两岸城市景观规划设计。加大对违规建设行为的查处力度，切实维护城市规划的严肃性和权威性。

加强基础设施建设。继续抓好西北环高速路、滨河东路延伸、窊流路、涧河路、旧晋祠路、迎新南三巷、解放南路、长治路、晋祠景区整治、城西水系建设等重点工程。实施动物园搬迁、森林公园二期、南北沙河治理等工程；加快汾河治理二期工程的立项、设计；抓紧新区规划论证和市政中心前期准备工作，适时启动新区建设。抓好生活垃圾焚烧发电厂和压缩中转站的建设，进一步完善城市环卫基础设施。积极发展住宅和房地产业，加大危旧房改造力度，经济适用房和危旧房改造开复工120万平方米，竣工60万平方米。

深化城市环境综合整治。巩固城市环境综合整治成果，严厉打击各种破坏整治成果的行为。进一步提升综合整治档次，建设标志性景观。实施开化寺街、亲贤北街亮化工程。把综合整治向小街巷、居住区和社区延伸，全面完成小街小巷的综合整治任务，彻底改变脏乱差状况，为广大市民创造良好的生活环境。对机场、车站、宾馆周边进行整治和美化，创造一流的服务环境和条件。继续实施“畅通工程”，改善交通状况。大力实施绿化工程，加强生态建设，高标准、高质量完成新晋祠路、太榆路等出口路的绿化，抓好胜利桥西两环绿化美化，完成30条新拓宽道路绿化任务，搞好社区绿化，加大居民区园林、绿地建设力度，绿化覆盖率、绿地率分别增加一个百分点，人均绿地增加一平方米。坚持不懈治理污染、改善环境。创新环保“五大工程”，全面推行清洁生产，开展清洁能源行动，积极倡导循环经济，搞好排污交易试点，严惩环境违法行为，市区空气质量二级以上天数达到180天，主要污染物指标下降15%以上。

加大经营城市力度。着力在城市资源的有效利用、城市资产的滚动增值、城市建设资金的有效投入上下功夫。创新城市土地收购储备制度，所有经营性用地一律公开出让，全年国土收益超过去年水平。加快城市公共服务行业市场化步伐，建立政府特许经营制度和公开招投标制度，吸引外国资金、民间资金参与供水、供气、供热、污水处理、垃圾处理等市政公用设施建设和经营。对市政设施、园林绿化、环境卫生等非经营性设施的日常养护，通过公开招标发包方式，面向社会选择作业单位或承包单位，提高养护效率和质量。

搞好“城中村”改造试点。以中心区、商业区、开发区、旅游景区“城中村”改造为重点，全市统一部署，各城区组织实施，在试点的基础上逐步推开，争取用3年时间完成主城区“城中村”改造任务。

加强城市长效管理。进一步完善城市管理体制，按照权随责走、费随事转的原则，强化城区、街道、社区管理城市的职能，把责任落实到基层。市有关部门要把更多的时间和精力放到搞好规划和加强监督检查上来，不断提高管理水平。搞好城市管理综合执法试点，切实加强执法队伍建设，做到严格执法、文明执法、规范执法。

（三）全面繁荣农村经济，加快农村小康进程

我市总体上已经达到小康水平，但这种小康是低水平、不全面、不平衡的小康，尤其是城乡之间差距较大，少数农民仍处于贫困状态。我们要把建设小康社会的重点放在农村，更多地关注农村、关心农民、支持农业，增加农民收入。

发展城市农业。以“优质、高产、高效、生态、安全”为核心，进一步调整农业结构，促进农业集约化经营，逐步把蔬菜、干鲜果、花卉、中药材等特色种植、养殖业培育成有规模、有市场、有品牌的主导产业。蔬菜播种面积39万亩以上，产量133万吨。推进名特果品示范园区建设，优质果品率达到70%以上。中药材种植面积4万亩，增长20%以上。大力发展畜牧业，牛奶产量增长15%以上。积极推进安全农产品生产，完成30个无公害农产品认证，建成无公害农产品基地30万亩，发展无公害畜禽养殖50万头（只）。

推进农业产业化经营。以建设全省农产品加工中心城市为目标，突出特色，做大规模，着力培育一批销售亿元以上的农产品加工龙头企业，扩大乳制品、肉制品、果菜、优质粮和中药材加工能力，农产品加工产值达到25亿元，增长25%。加强农村中介组织和经纪人队伍建设，提高农业市场化、组织化程度，带动农户20万户，增长11%。

巩固扩大退耕还林成果。按照“谁退耕、谁造林、谁经营、谁受益”的原则，鼓励农民群众和社会各界承包宜林荒山，购买“四荒”，植树造林。高标准、高质量完成今年退耕还林45万亩的任务。加快环城林带建设，植树造林15.6万亩，当年形成规模，构建省城绿色屏障。

加快小城镇建设。以县城和有条件的建制镇为基础，逐步在城市周围形成一批特色小城镇，带动周边农村快速发展，促进农民向非农产业和城镇转移。调整乡镇企业布局和结构，引导乡镇企业向小城镇集中，增加值和上缴税金分别增长20%以上。

加强农村扶贫开发工作。继续实施“五大增收致富工程”，新增脱贫人口3.3万人。全面推进乡村公路建设，积极筹措资金，实行“以奖代补”政策，今年完成1200公里，基本实现村村通油路、水泥路，解决农民出行难问题。有计划进行移民并村工作。继续实施饮水解困工程，解决8万人饮水困难或水质不达标问题。认真搞好农村税费改革，切实减轻农民负担，坚决打击各种坑农、害农行为。

（四）扩大社会需求，促进对外开放

在积极扩大国内市场的同时，大力开拓国际市场，加大招商引资力度，实施更高层次、更宽领域的对外开放，“让太原走向世界，让世界了解太原”，以开放促改革促发展，不断增强太原的国际经济竞争力。

积极开拓消费市场。着力发展新兴服务业，培育假日、健身、文化、教育、住房、信息等新的消费热点。积极推进特许经营、连锁经营、电子商务等新型经营方式，加快现代商贸流通业发展。以华宇集团、桥西农副产品批发市场为龙头，培育年销售10亿元、利税5000万元以上的大型骨干企业。加强商贸旅游结合，抓紧柳北、桥头街、钟楼街等特色商业街区的开发改造。积极发展物流配送，统筹规划，协调推进，逐步形成主业突出、核心能力强、覆盖市区的物流中心和配送中心。完善农村销售服务网络，开拓农村市场。

努力扩大出口。积极推动市场和出口产品多元化，巩固扩大美、日、韩、欧等传统市场，努力开拓拉美、中东、非洲和俄罗斯等新兴市场。优化出口结构，在增加焦炭、铝锭、金属镁等传统产品出口的基础上，提高机电产品和成套设备的出口份额。重点帮助大型企业、民营企业、高新技术企业取得外贸出口经营权，形成新的出口增长点，实现出口高增长。

提高利用外资的质量和水平。以“招商旅游年”和纪念建城2500年为契机，加大招商引资力度。以潜力产品、高新技术、基础设施、农业产业化、旅游文化等为重点，精选一批科技含量高、关联度强、效益好的重点项目开展招商活动，吸引跨国公司和国内知名企业对我市企业进行改造重组。进一步落实招商引资责任制，抓好已签协议、意向项目的跟踪督办，提高合作成功率，力争合同利用外资再翻一番。

（五）强化财税管理，深化财政改革

积极推进各项财税改革，扩大税源，调整支出结构，充分发挥财政宏观调控职能，促进全市经济持续快速健康发展。

加大税收征管力度。以“金税工程”为龙头，加强税收征管信息化建设，推广税控装置，搞好收入动态监控，提高税收征缴入库率。坚持依法治税，严厉打击偷税、漏税、逃税、抗税等违法行为，做到应收尽收，不虚收探收。把财源建设与经济结构调整紧密结合，大力培植以新兴产业、优势产业和骨干企业为重点的新财源，确保财力稳步增长。

优化支出结构。积极建立公共财政，创新理财机制，按照“一要吃饭，二要建设”的原则，集中财力保重点、办大事。优先安排工资性支出和社会保障经费，确保全市各级机关、事业单位工资和社保金按时足额发放，社保支出增长22.8%。逐步提高财政对公共需要的投入能力，支农支出增长12%，科技三项费增长13.9%，教育支出增长13.7%。加快财政从竞争性领域退出步伐。牢固树立过紧日子思想，节约使用财政资源，反对铺张浪费。除工资性支出、社保支出、法定支出保持增长外，其余一般性支出实行零增长。加强财政性资金管理，强化审计监督，严厉查处各种违反财经纪律的行为，防止财政资金流失。

深化财政改革。继续完善部门预算，强化预算约束力。全面推行政府采购，80%政府性采购资金通过集中采购实施。开展国库集中收付试点，建立以国库单一账户体系为基础、资金缴拨以国库集中收付为主要形式的财政国库管理制度。完善以会计集中核算为主的会计委派制度，积极推进“金财工程”建设，提高财政管理信息化水平。

（六）大力实施“科教兴市”战略，加快推进社会事业改革

适应社会主义市场经济发展要求，加大社会事业改革力度，打造文化旅游强市，提高全市人民的科学文化素质和健康素质，促进人的全面发展。

推进科技创新和应用。继续搞好与全国、全省知名高校的全面合作，促进科研成果在我市转化。积极引导大中型企业与科研院所紧密合作，建立企业技术中心，形成一批具有自主知识产权的科技成果。创新科技管理体制，积极推行重大项目招投标制度。

提高教育水平。进一步巩固“两基”成果，继续深化教育改革，加强教学管理，推进素质教育，提高教育质量，高考升学率保持全省领先水平。整合教育资源，调整教育结构，抓好标准化和示范高中建设，启动教育园区建设。大力推进教育信息化，完成500所农村学校远程教育卫星网建设，基本完成“校校通”、教育城域网和教育资源库一期工程。加强社区教育和职业教育，发展继续教育，逐步构建终身教育体系。积极推进中央、省属企业学校剥离工作。加强教育资产管理，防止教育资产流失。

塑造文化、旅游精品。按照政府引导、市场运作的原则，搞好“招商旅游年”和纪念建城2500年系列活动，加大文化遗产的开发、保护和宣传力度，打造一批晋阳文化品牌和旅游精品，提升城市文化形象。继续抓好太原申报全国历史文化名城和晋祠申报世界文化遗产工作，力争取得突破性进展。大力发展文化产业，抓好报业集团的组建和广播电视总台的运作。筹建晋阳文化艺术中心。推出一批有太原特色、质量较高的文艺作品。加强文物保护，整合文物和旅游资源，充分挖掘旅游文化内涵，重点对晋祠、双塔寺、崛围山景区进行整治，完成拱极门等一批文物古迹的修复、恢复工程。规范旅游市场，加大促销力度，尽快把旅游业培育成我市支柱产业。全年接待海内外游客1200万人次，旅游总收入40亿元，分别增长15%和12%以上。

发展卫生、体育事业。引深医疗卫生改革，进一步规范药品、医用材料集中招标采购，推进医院后勤服务社会化。普及社区卫生服务机构，覆盖率达到80%以上。建立和完善以大病统筹为主的新型农村合作医疗制度，提高农村卫生保健服务质量。加强体育设施建设，结合园林布局，增加市民健身场地，完善全民健身体系。办好第五届全国城运会自行车比赛，争取在城运会上取得好成绩。

强化人口与计划生育工作。坚持发展经济与控制人口一起抓，加强计生干部队伍建设和计划生育信息建设，夯实基层基础工作。深入开展宣传教育，推进依法管理，搞好优质服务。加大政策扶持力度，确保计划生育工作所需经费。严格实行目标责任制，严肃查处计生违法违纪行为，努力提高计划生育工作水平。

深化人事制度改革。稳步推进事业单位人事制度改革，加快事业单位企业化管理步伐。加强公务员队伍建设，提高公务员整体素质。制定落实人才优惠政策，培育完善人才市场，努力形成有利于培养人才、吸引人才、用好人才的新机制。

加强社区建设。建立覆盖全市社区的网络平台，实施100个“星光计划”项目，鼓励、支持社会力量投资，改善社区居委会办公和服务条件。规范社区居委会的职责和功能，加强干部队伍建设，提高社区自治水平，使社区更快更好地承接社会保障、社区服务、社会管理功能。以保洁保绿、家政服务、卫生保健、托老托幼、物业管理为重点，发展壮大社区服务业，扩大社区就业，方便居民生活。力争把三分之一以上的社区建成管理有序、服务完善、环境优美、治安良好、生活便利、人际关系和谐的示范性现代化社区。

（七）开发就业岗位，完善社会保障体系

增加就业是今年政府宏观调控的主要目标。要努力改善就业环境，积极开发就业岗位。扩大社会保障覆盖面，建立健全与经济发展水平相适应的社会保障体系。

广开就业门路。实施就业优先战略，千方百计扩大就业渠道，重点解决好下岗、失业人员再就业问题，再就业率达到45%以上。制定出台政府购买公益性岗位安置就业困难人员、完善就业服务体系等政策措施。落实再就业各项优惠政策，鼓励下岗失业人员自谋职业、自主创业。政府引导与市场运作相结合，建设再就业培育性经营基地。充分发挥中小企业、民营企业、社区服务业吸纳就业的主渠道作用，扶持发展各种形式的弹性就业，全年开发就业岗位12.6万个，安置“4050”人员4000名。加强劳动力市场和就业服务体系建设，建立覆盖全市的四级劳动保障工作平台，再就业培训率达到70%以上。强化劳动管理，规范企业用工行为，保护职工特别是农民工的合法权益。

完善社会保障。继续巩固“两个确保”，抓好“三条保障线”的衔接。积极采取措施，引导下岗职工出中心，向失业保险并轨。以私营、个体等非公有制经济组织为重点，扩大养老、失业保险覆盖面，非公有制经济的参保率达到80%以上。加快企业离退休人员管理服务进社区的进程，提高社会化管理程度。完善和推进城镇职工基本医疗保险改革，出台机关、事业单位医疗保险补助办法和离休人员医疗保险实施办法，研究制定困难企业和民营企业参保的政策措施。严格执行国家低保政策，加强资金统筹，实现动态管理下的应保尽保。

建立长效救助机制。构建临时救助、生活补助和社会捐助相结合的社会救助模式，在重点保障困难群众基本生活的基础上，重视解决城市特殊困难家庭在住房、子女就学、医疗等方面遇到的问题。做好优抚安置工作，切实解决老红军、老复员军人、伤残军人、烈属等重点优抚对象的生活、住房、医疗问题。落实义务兵家属优待政策，做好转业复员退伍军人安置工作。发展社会福利事业，搞好救灾救济，为灾民、贫困户和农村五保户解决实际困难。动员社会力量，进家入户，帮助困难群众摆脱困境，使送温暖、献爱心活动经常化、制度化、社会化。

（八）加强精神文明和民主法制建设，营造良好社会氛围

引深创建全国文明城市活动，贯彻落实《公民道德建设实施纲要》，加强社会公德、职业道德和家庭美德教育。增强全民信用意识，逐步建立和完善以政府为主导、企业为主体的社会信用体系，努力打造信用太原。各级政府要认真执行人大及其常委会的决议、决定，自觉接受人大及其常委会的依法监督，接受政协、民主党派、工商联及各社会团体的民主监督，接受人民群众和社会舆论监督，做好人大议案、建议和政协提案的办理工作。支持工会、共青团、妇联等人民团体开展工作。加强双拥工作，争创双拥模范城“五连冠”。全面落实党的民族宗教政策，依法管理民族宗教事务。加强城乡基层政权和群众性自治组织建设，搞好第六届村委会换届工作。进一步推进企务公开、村务公开，不断扩大基层民主。认真接待群众来信来访，切实解决群众反映强烈的热点、难点问题。抓好“四五”普法教育，提高全民法律素质。加强社会治安综合治理，深入开展严打整治斗争，严厉打击各种刑事犯罪、经济犯罪和邪教组织的非法活动，全力维护社会稳定。净化文化市场，扫除“黄赌毒”等社会丑恶现象。加强安全生产监督管理，防止重特大事故的发生。整顿规范市场经济秩序，打击制假售假行为，创造公平、有序、健康的市场环境。新闻出版、档案、气象、地震、侨务、对台、人防、国防后备力量建设、地方志、老龄、残疾人等工作，也要有新发展、新提高，取得新成绩。

（九）深化行政管理体制改革，进一步加强政府自身建设

以建立行为规范、运转协调、公正透明、廉洁高效的行政管理体制为目标，转变政府职能，创新管理方式，提高工作效率，确保完成各项任务。

更新思想观念。进一步解放思想，与时俱进，努力在发展思路、工作方法上有所创新，在抓住机遇、加快发展上有所作为。不断增强学习意识，努力建设学习型政府，提高干部队伍的综合素质。不断增强创新意识，按照“三个有利于”标准，打破常规，冲破常态，奋发进取，争先争上。不断增强机遇意识，着力在完善机制、强化服务上多下功夫，使各项工作更富有成效。不断增强法制意识，加大行政执法监督力度，坚持依法行政，从严治政。

转变政府职能。深化政府机构改革，科学规范部门职能，切实解决越位、缺位和错位问题，真正把政府职能转到经济调节、市场监管、社会管理和公共事务上来。深化行政审批制度改革，在减少审批事项、下放事权的基础上，规范审批行为，健全监管措施。抓好政务大厅的运行和管理，充分发挥窗口示范作用。推行“阳光政务”，拓展和规范政务公开的内容和范围。加大“电子政府”建设力度，加快政务信息化步伐，不断提高行政效率。

改进工作作风。坚持相信群众、依靠群众、关心群众、服务群众，切实做到心为民想，情为民系，利为民谋，权为民用，把想群众所想、急群众所急、办群众所盼，化为最经常、最本职的工作，切切实实解决人民群众在日常生活中遇到的种种不便和困难。建立健全各级政府和领导干部联系群众制度，拓宽人民群众反映问题和意见的渠道。牢记“两个务必”，弘扬艰苦奋斗精神，反对讲排场、比阔气，坚持勤俭办一切事情。发扬求真务实、团结协作的作风，切实改变推诿扯皮、办事拖拉、敷衍塞责的不良现象。加强廉政建设，深入开展反腐败斗争，坚决纠正部门和行业不正之风，努力从源头上、机制上、制度上预防和治理腐败。

落实目标责任。围绕率先发展，把目标和任务层层分解，落实到基层、落实到人头，强化责任约束，形成一级抓一级、一级对一级负责，环环相扣、层次分明的责任体系，严格考核，奖惩兑现，调动方方面面的积极性，把全市上下的力量凝聚到实现率先发展第一要务上来。

各位代表！时代赋予我们重任，人民寄予我们厚望。让我们更加紧密地团结在以胡锦涛同志为总书记的党中央周围，忠诚实践“三个代表”重要思想，在省委、省政府和市委的正确领导下，同心同德，扎实工作，开拓前进，奋发图强，圆满完成率先发展的各项任务，谱写全面建设小康社会的新篇章！
